# Complications Using Bioabsorbable Cross-Pin Femoral Fixation: A Case Report and Review of the Literature

**DOI:** 10.1155/2011/349230

**Published:** 2011-09-25

**Authors:** Saqib Hasan, Samir Nayyar, Ikemefuna Onyekwelu, Kunal Kalra, Soterios Gyftopoulos, Laith M. Jazrawi

**Affiliations:** ^1^Department of Orthopaedic Surgery, NYU Hospital for Joint Diseases, New York, NY 10003, USA; ^2^Department of Radiology, NYU Langone Medical Center, New York, NY 10016, USA

## Abstract

The use of bioabsorbable cross-pin transcondylar fixation has remained a viable option for femoral fixation in anterior cruciate ligament reconstruction. Although numerous biomechanical studies have demonstrated high fixation strength and minimal slippage with use of this method of fixation, there have been increasing reports of a variety of clinical complications associated with these implants. We reviewed the literature for all complications associated with the Bio-TransFix implant and present a case report of a patient status after ACL reconstruction using Bio-TransFix cross-pin femoral fixation with iliotibial band friction syndrome from a broken cross-pin four month post-operatively.

## 1. Introduction

Anterior cruciate ligament injuries are a significant cause of disability in active individuals with an estimated incidence of 80,000–200,000 ACL injuries occurring in the United States each year [1, 2]. With approximately 100,000 graft reconstructions performed in the United States annually [3], there are a variety of techniques available with respect to the type of fixation and choice of graft tissue. While the choice of graft tissue has received considerable attention in the context of patient outcomes, the method by which a graft is fixed is of paramount importance in dictating the robustness of the graft construct; the fixation device may represent the weakest link in the ACL reconstruction. Optimal graft fixation should be structurally secure, recapitulate normal tendon healing and allow for the graft construct to replicate the biomechanical properties and biological composition of the native ligament. It is imperative that the mechanical fixation device offers these properties until full incorporation of the graft and biological fixation have occurred. A study [4] evaluating tendon-to-bone healing in a dog model demonstrated that graft fixation strength must withstand the stresses applied to the native ACL during the healing interval, which can require 6 weeks for bone incorporation and 12 weeks for soft tissue incorporation. The strength of the fixation and not the strength of the graft is the weak point during the early postoperative period, hence, the ideal graft fixation would allow for aggressive postoperative rehabilitation with goals of immediate weight-bearing, full range of motion and return to athletic activity [5–7]. Although there are many different aspects of mechanical graft fixation, given our case presentation, we focus on femoral fixation of softtissue hamstring grafts using the Bio-TransFix implant for transcondylar cross pin femoral fixation.

## 2. Case Report

**Figure 1:**
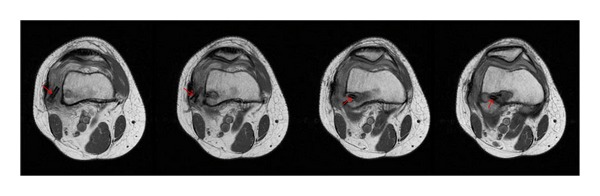
Axial PD fat suppressed T2-weighted images of a right knee in a patient with prior anterior cruciate reconstruction demonstrate a fractured Bio-TransFix cross pin (red arrows).

**Figure 2:**
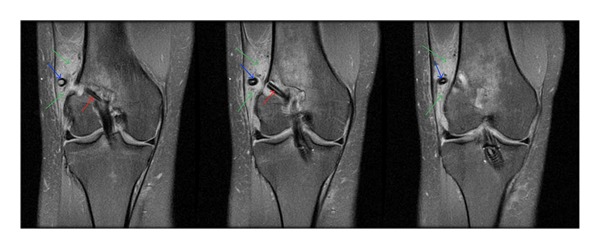
Coronal fat suppressed T2-weighted images demonstrate that the proximal portion of the pin has backed out of the distal femur and is found within the adjacent soft tissues where it is surrounded by edema (blue arrows) and a thickened iliotibial band (green arrows). The distal portion of the pin (red arrows) and ACL graft are intact.

A 29-year-old female presented to our orthopaedic service following a twisting injury during martial arts, where she heard her right knee pop and experienced immediate pain and swelling in her knee. Physical examination demonstrated +2 Lachman and +1 pivot in her right knee. MRI confirmed full thickness ACL tear, as well as medial and lateral meniscal pathology with a lateral meniscal tear. Arthroscopic ACL reconstruction of the patient's right knee was performed using allograft and the Bio-TransFix system (Arthrex; Naples, FL). Following drilling of the tibial tunnel and guide placements, the Bio-TransFix guide and pin were inserted and secured in the graft. An 8–10 mm IntraFix screw and sleeve construct were then inserted at this time. The graft was noted to be taut, and full extension showed no impingement both on the PCL and on the notch. There were no intraoperative complications. At six weeks after the ACL reconstruction procedure, clinical examination revealed some mild wound breakdown at the tibia and a prominence over the lateral incision, through which the cross-pin was inserted. The patient's range of motion was from 0 to 90 at this time. Lachman test was negative. The patient had no symptoms of instability. At four months after ACL reconstruction, the patient was doing well, but was still having pain over the lateral incision. An MRI of the right knee demonstrated a broken, displaced cross-pin. The proximal portion of the pin was displaced into the adjacent soft tissues and surrounded by extensive edema and a thickened iliotibial band (Figures 1 and 2). The ACL graft was intact.

**Figure 3:**
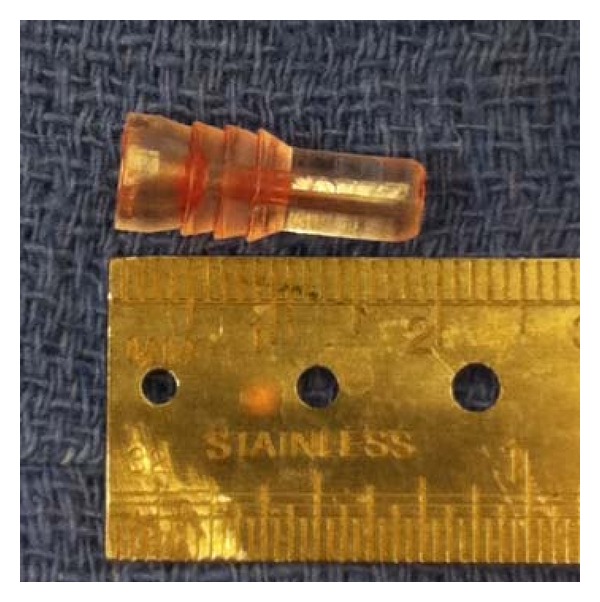
Broken tip of Bio-TransFix implant.

The patient continued to have pain and elected to undergo surgery. Under general anesthesia, an incision over the previous one was performed. The IT band was split and the underlying soft tissue palpated. The Bio-TransFix pin was found to be broken and was then removed with a clamp (Figure 3). Six months after the surgery, patient is doing well and has gone back to martial arts with no restrictions. Patient clinically has full range of motion in her right knee and a negative Lachman test. Patient reported complete resolution of symptoms following pin removal.

## 3. Discussion

Mechanical fixation can be categorized as direct or indirect. Direct fixation, as seen with interference screws, staples and spiked washers, refers to compression of the soft tissue to allow direct contact healing between the graft and the bone surface without the development of a fibrous interzone normally seen in nonanatomic fixation methods. [8]. Indirect fixation suspends the graft in the bone tunnel and can be further divided into cortical, cancellous, or cortical-cancellous suspension. Cortical suspension devices, such as EndoButton, suspend the graft using a hardware component placed on the anterior-lateral cortex of the distal femur; cancellous suspension systems suspend the graft from a screw that is fixed into the cancellous bone of the femoral metaphysis. Finally, cortical-cancellous suspension systems, such as cross-pin fixation, utilize a transcondylar suspension pin placed perpendicular to the graft. According to numerous studies [9–11], cross-pin femoral fixation has been shown to provide high fixation strength and sufficient resistance against slippage. In a biomechanical study comparing nine different femoral fixation devices with various fixation mechanisms, cortical-cancellous suspension fixation achieved with transcondylar devices seemed to offer the best results in terms of graft elongation, fixation strength, and stiffness when compared to fixation via compression, expansion, cortical suspension, and cancellous suspension [12].

Metal cross-pin femoral fixation was first described by Clark et al. [13] as an excellent method of fixation due to its superior strength and stiffness in comparison to other femoral fixation devices. Clark and colleagues early work on cross-pin femoral fixation led to changes in design due to concerns of fatigue fracture at the graft implant interface. Development of bioabsorbable implants using poly-L-lactic acid (PLLA) allowed for the further advantages of undistorted MRI imaging and uncompromised revision surgery. In a biomechanical study, Milano et al. found [12] that both bioabsorbable and titanium TranFix implants offer comparable results in terms of elongation, fixation strength and stiffness. 

The Bio-TransFix femoral fixation device places a bioabsorbable pin across the femur traversing the femoral tunnel for secure femoral fixation of the graft construct acting as transverse suspension bar perpendicular to pullout forces. As demonstrated by Ahmad et al. in a biomechanical study using 33 porcine femora to evaluate femoral soft tissue fixation, the Bio-TransFix cross-pin fixation provided high fixation strength and sufficient resistance against slippage in comparison to the conventional interference screw fixation and other similar devices [9]. In a pilot randomized controlled trial of femoral fixation of a hamstring autograft in 30 patients with 13-month followup, Capuano et al. [14] demonstrated no significant change in side-to-side laxity and IKDC scores between biointerference screw (Arthrex Inc, Naples, FL) and Bio-TransFix femoral fixation. Numerous other studies have demonstrated that the Bio-TransFix and its nonbioabsorbable equivalent (TransFix) appeared to be either superior or comparable to other available femoral fixation systems [12, 15–17].

While biomechanical studies appear to support the use of the Bio-TransFix and TransFix femoral fixation devices, there is a paucity of clinical studies with long-term followup. One controlled prospective randomized study with 2-year followup demonstrated no statistically or clinically relevant differences between cross-pin femoral fixation versus metal interference screw fixation [18]. Similarly, other clinical studies have not demonstrated any statistically significant advantages of using cross-pin femoral fixation over various other fixation devices [19, 20]. A recent meta-analysis examined optimal femoral fixation in hamstring autografts by comparing interference screw versus non-interference screw fixation using surgical failures and IKDC scores as common endpoints [21]. It was shown that non-interference screw methods of fixation such as Bio-TransFix, TransFix, RigidFix (DePuy Mitek, Norderstedt, Germany), and EndoButton (Smith and Nephew Inc, Andover, MA) showed no difference between post-operative functional outcomes when compared to interference screw fixation. However, it was noted that non-interference screw fixation had higher rates of surgical failures in comparison to interference screw fixation.

### 3.1. Complications Associated with Bioabsorbable Cross-Pins

Despite the ubiquitous use of cross-pin femoral fixation, there have been numerous intraoperative as well as postoperative complications reported in the literature. Cross-pin femoral fixation devices such as RigidFix have been associated with a variety of complications ranging from lateral pin slip [9] and tunnel widening [22] to implant protrusion and breakage of bioabsorbable cross-pins [23–27]. The literature also documents similar complications associated with the Bio-TransFix device. Kokkinakis et al. [28] demonstrated three cases of iliotibial band friction syndrome after lateral pin translation of the Bio-TransFix implant with resolution of symptoms after implant removal. All three cases had developed symptoms within the first 2-3 months post-operatively. There was intraoperative confirmation of the correct implant depth and good coverage of the pin head into the femoral cortex in each case. Misral et al. [29] presented one case of a patient who presented 2-month post-operatively with knee pain, effusion, and decreased range of motion with maximal tenderness along the medial aspect of the patella secondary to intra-articular pin translation. The implant was found to be protruding into the medial retinacular area with resolution of symptoms after arthroscopic removal of the terminal end of the protruding pin. Marx and spock [30] presented both lateral and medial pin migration in two patients who presented with discomfort 4-5 months postoperatively. In both cases a prominence was felt at the distal aspect of the femur corresponding to the migrated implant. The lateral pin was found to have been proud by 4 mm and was impacted with a punch. The medial pin was found to be proximal to the superomedial aspect of the patella with MRI confirmation of intra-articular protrusion. The terminal tip of the pin was removed arthroscopically. It is important to note, however, that the complications reported were potentially due to technical error secondary to inappropriate positioning of the femoral tunnels [31]. Arriaza et al. [32] reported a novel complication of stress fractures of the medial femoral supracondylar area in two professional athletes who presented at 2 and 16 weeks post-operatively. However, the authors felt that these complications were potentially secondary to an accelerated rehabilitation program in conjunction with the medial cortical hole of the Bio-TranFix guide acting as a stress riser.

There has been only one previously reported case of Bio-TransFix pin breakage with resultant clinical complications. Pelfort et al. [33] described the development of iliotibial band friction syndrome in two patients 3 months post-operatively. MRI confirmed breakage and lateral migration of the implant tail in both cases with resolution of symptoms after removal via a minimal lateral approach. Our case report is the second reported case of breakage of the Bio-TransFix pin with ensuing clinical symptoms. It is important to note that breakage of bioabsorbable transcondylar pins has been reported in numerous studies as a relatively common complication [23, 26, 34]. Cossey et al. [34] reported on 49 patients who underwent quadrupled semitendinosus ACL reconstruction using the Bio-TransFix implant with an accelerated rehabilitation program. Post-operative clinical assessment and MRI found 16% of implants to be fractured or deformed, many during the period of biological graft incorporation. Interestingly, all patients with fractured or deformed pins had no symptoms or signs of instability and successfully returned to preinjury sporting activities. A recent retrospective imaging study [26] looked at RigidFix bioabsorbable cross-pin fixation in 202 patients with an average followup of 26 months postoperatively. Using MRI, Studeler et al. found fractured cross pins in 35 patients (17%), breach of the posterior femoral cortex in 57 patients (28%), and migration of fractured pin fragments in 12 patents (6%). Again, there was no correlation between fractured pins and graft nor was there any clinical correlation between fractured pins and instability. These findings contrast with data reported by Choi et al. [23] in their analysis of 31 patients who underwent ACL reconstruction using RigidFix bioabsorbable cross-pin fixation. Using MRI, the authors found that 38.7% of the RigidFix implants fractured 6 months postoperatively. The authors found significant correlation between laxity and fractured cross-pins as measured using an arttrometer. However, the authors reported no difference in Lachman and pivot-shift test data between fractured and nonfractured pins two years postoperatively. While complications arising from the breakage of the Bio-TransFix have been scarcely reported, there are case studies documenting clinical complications arising from the breakage of the RigidFix bioabsorbable cross-pin, including a chondral lesion on lateral femoral and tibial condyle [35] as well as a broken pin in the posterolateral compartment of the knee causing a painful catching sensation during leg extension [25].

### 3.2. Technical Recommendations

Although the literature has not shown a definitive correlation between intraoperative technique and complications arising from bioabsorbable cross-pins, we feel that there are numerous technical considerations with regard to this type of femoral fixation. For the Bio-TransFix implant, our recommendations extend to taking measures to ensure that the implant is not broken intraoperatively as well as confirming complete seating of the implant once inserted. In the case report presented, it is plausible that our implant malfunction was the result of intra-operative difficulties. Our experience with this implant has shown that, during insertion, the wire or implant may break if there is an alignment mismatch. The bioabsorbable implant has a low resistance to tensile forces, and it may break if it is not inserted parallel to the nitinol wire. To prevent breakage of the implant, we recommend that retrieval of the nitinol wire from the lateral to medial femoral cortex be done in line with the drilled pin using a smooth, straight motion to avoid causing any kinks in the wire. The wire should be held as taut as possible in the same direction of the pin. Once the wire has been inserted, the implant should be tapped in the same orientation as the wire in a gradual stepwise manner. Kocher clamps should be attached to both sides of the wire, with one clamp attached near the implant inserter approximately 1 cm from the distal end of the slot in the inserter. The wire should be pulled from the medial side until the clamp touches the distal end of the slot and can no longer be advanced. The implant should then be gently tapped along the wire, again taking care to ensure proper orientation. This will recreate the distance between the clamp and the distal end of the slot. The medial side of the wire should then be pulled until the clamp advances to the end of the slot. Repeat these steps until the inserter bottoms out to the marking (on the inserter) near the skin as noted before. It is important not to tap beyond this marking as the implant will no longer advance and may break inside the bone resulting in post-operative migration. Steps should also be taken to ensure that there has been complete seating of the implant. The measurements off the reamer, broach, and the implant inserter should all be confirmed to be the same. Once the implant has been inserted, the lateral cortex should be digitally palpated.

## 4. Conclusion

Although the use of the bio-absorbable cross-pins has received considerable attention as a means of femoral fixation for anterior cruciate reconstruction, case series, and imaging studies have shown that a significant number of cross-pins can fracture and migrate with a potential for resultant clinical complications. Due to the asymptomatic or nonspecific nature of these complications, imaging studies are an integral part of patient management. Our case report illustrates the common phenomenon of implant breakage with a relatively rare complication of iliotibial band friction syndrome. It is possible that this complication was due to a combination of inherent implant failure and intra-operative difficulties. Given our experience and review of the literature, we feel that is it critical to be aware of the intra-operative difficulties that can be associated with this implant and encourage a low threshold for suspicion of pin breakage and migration when patients present with clinical complaints after ACL reconstruction. While the use of X-rays may serve as an initial screening modality to demonstrate pin breakage in these patients, the use of magnetic resonance imaging serves as a better imaging modality as it can not only diagnose fractured cross-pins but also reveal any surrounding soft tissue findings such as edema, IT band syndrome as well as provide information on state of the ACL graft. Patients with complications associated with cross-pin breakage and migration will often have complete resolution of symptoms once the offending fragment is surgically removed.
